# NMR backbone assignments of the tyrosine kinase domain of human fibroblast growth factor receptor 3 in apo state and in complex with inhibitor PD173074

**DOI:** 10.1007/s12104-018-9814-7

**Published:** 2018-03-26

**Authors:** Domenico Sanfelice, Hans Koss, Tom D. Bunney, Gary S. Thompson, Brendan Farrell, Matilda Katan, Alexander L. Breeze

**Affiliations:** 1 Institute of Structural and Molecular Biology, Division of Biosciences, University College London, Gower St, London, WC1E 6BT UK; 2 Astbury Centre for Structural Molecular Biology, Faculty of Biological Sciences, University of Leeds, Leeds, LS2 9JT UK; 3 Present Address: Wellcome Trust Biomolecular NMR Facility, School of Biosciences, University of Kent, Canterbury, CT2 7NZ UK

**Keywords:** Fibroblast growth factor receptor 3, Tyrosine kinase inhibitor, NMR resonance assignment, Cancer, Angiogenesis

## Abstract

Fibroblast growth factors receptors (FGFR) are transmembrane protein tyrosine kinases involved in many cellular process, including growth, differentiation and angiogenesis. Dysregulation of FGFR enzymatic activity is associated with developmental disorders and cancers; therefore FGFRs have become attractive targets for drug discovery, with a number of agents in late-stage clinical trials. Here, we present the backbone resonance assignments of FGFR3 tyrosine kinase domain in the ligand-free form and in complex with the canonical FGFR kinase inhibitor PD173074. Analysis of chemical shift changes upon inhibitor binding highlights a characteristic pattern of allosteric network perturbations that is of relevance for future drug discovery activities aimed at development of conformationally-selective FGFR inhibitors.

## Biological context

Four fibroblast growth factors receptors (FGFR1–4) are known to interact with several FGFs (22) to regulate critical cellular processes (Beenken and Mohammadi 2009; Brooks et al. 2012). Binding of FGFs leads to dimerization of FGFRs and phosphorylation of specific intracellular domain tyrosine residues; this is the first event of many signalling cascades regulating cell proliferation, differentiation and migration (Eswarakumar et al. 2005; Klint and Claesson-Welsh 1999). Dysregulation of these signalling cascades leads to several developmental syndromes and a broad range of human malignancies (Dieci et al. 2013; Katoh 2016). Structural and molecular dynamic properties of FGFRs are the subject of extensive study, as part of a mission to understand physiological and aberrant activation mechanisms as well as drug action (Chen et al. 2017; Huang et al. 2013; Klein et al. 2015; Kobashigawa et al. 2016; Patani et al. 2016; Perdios et al. 2017). To date, many kinase inhibitors have been developed and some have reached clinical trials (Zhang et al. 2009). PD173074 (PD) was developed as an ATP-competitive inhibitor for FGFR1 (Mohammadi et al. 1998) and it also binds tightly to FGFR3 (Grand et al. 2004). Here, we present the backbone amide NMR resonance assignments for FGFR3 kinase domain in ligand-free and PD-bound states. Comparison of free and bound states provides useful information regarding the binding site and will prove helpful in the design of next-generation kinase inhibitors.

## Methods and experiments

### Protein expression

The wild-type FGFR3 kinase domain (amino acids 455–768) was cloned into either pOPINS (OPPF, Oxford, UK) or pJ821 (DNA2.0, Menlo Park, USA) using In-Fusion cloning (Clontech, Mountain View, USA). Plasmids were transformed into C41 (DE3) cells harbouring a co-expression plasmid, pCDF-Duet, expressing lambda phosphatase under an IPTG-inducible promoter. The recombinant kinase domain was expressed as a His-tag fusion protein after induction with 0.1 mM IPTG (for pOPINS) or 1 mM rhamnose and 0.1 mM IPTG (for pJ821) for around 66 h at 16 °C.

Uniform stable isotope labelling was achieved by growing cells in D_2_O-based M9 minimal medium supplemented with ^15^N-ammonium sulfate (^15^NH_4_Cl) together with U-[^1^H,^13^C]-glucose (Cambridge Isotope Laboratories or Sigma-Aldrich) as sole nitrogen and carbon sources, respectively. Deuterium adaptation was achieved using minimal medium agar plates: each plate was allowed to grow for 48 h at 37 °C. Cultures were grown in baffled 2 L flasks for 2 h at 37 °C and then 4 h at 15 °C. Amino-acid-selectively labelled samples were prepared by growth in media containing all amino acids at a concentration of 1000 mg/L, but depleted in the target unlabelled amino acid, which was supplemented in the required labelled form (Sigma-Aldrich) at 100 mg/L immediately prior to induction. Amino-acid-selectively unlabelled samples were prepared by growth in M9 minimal media containing ^15^NH_4_Cl and an excess of unlabelled specific amino acid.

### Protein purification

Frozen pellets were resuspended in 20 mL of chilled Lysis Buffer (25 mM Tris–HCl, 250 mM NaCl, 40 mM imidazole, 10 mM benzamidine, 1 mM MgCl_2_, 100 µM CaCl_2_ and 100 µg/mL lysozyme, pH 8.0). Lysis was continued by the addition of 5 mL of a solution of 10% (v/v) Triton-X-100 and 1 K unit of bovine pancreatic DNAse I at 4 °C. Harvested clear cell lysates were loaded onto a 5 mL HisTrap column (GE Healthcare, Amersham, UK). Unbound proteins were washed out with His Buffer A (25 mM Tris–HCl, 500 mM NaCl, 40 mM imidazole, 1 mM TCEP, pH 8.0) and eluted with a 20-column volume gradient containing 500 mM imidazole. Eluted fractions were pooled together and the His-tag was cleaved using Ulp1 protease while dialyzing overnight against Dialysis Buffer (25 mM Tris–HCl, 1 mM TCEP, pH 8.0) and separated by a second HisTrap purification step. Unbound FGFR3 was injected on a 5 mL HiTrap Q (GE Healthcare, Amersham, UK) equilibrated in Q Buffer A (25 mM Tris–HCl, 20 mM NaCl, 1 mM TCEP, pH 8.0). Elution was achieved with 20 column volumes to 50% of Q Buffer B (25 mM Tris–HCl, 1 M NaCl, 1 mM TCEP, pH 8.0). Finally, fractions containing FGFR kinase domain were pooled and injected onto a Superdex 200 26/60 column (GE Healthcare, Amersham, UK) equilibrated with NMR buffer (50 mM PIPES-NaOH, 50 mM NaCl, 2 mM TCEP, 1 mM EDTA, pH 7.0). Monomeric FGFR3 kinase domain was concentrated in Vivaspin 10 kDa m.w.c.o. (Vivaproducts, Littleton, USA) concentrating units and quantified using a Nanodrop (Thermo Scientific, UK), using calculated molecular weight and extinction coefficients. Proteins were stored at between 5 and 20 mg/mL, after snap-freezing in liquid N_2_, at − 80 °C.

### NMR spectroscopy and data processing

Uniformly ^15^N,^13^C,^2^H-labelled, uniformly ^15^N-labelled, selectively-labelled and selectively-unlabelled samples of WT FGFR3, were prepared in 50 mM PIPES-NaOH, 50 mM NaCl, 5 mM TCEP and 1 mM EDTA (pH 7.0) containing 5% D_2_O. PD173074 was added from concentrated stock solutions prepared in DMSO where required. All samples were approximately 300 µL and between 77 and 230 µM concentration in 5 mm Shigemi tubes. NMR spectra were recorded at 298 K for ligand-free FGFR3 and 303 K for the PD complex, on Bruker Avance III or Avance III HD 800 or 950 MHz spectrometers equipped with TCI z-axis gradient Cryoprobes. Standard TROSY-detected triple-resonance experiments (Salzmann et al. 1998) and TROSY-detected HSQC experiments with water flip-back and WATERGATE pulses (Pervushin et al. 1998) were recorded as detailed previously (Bunney et al. 2015). ^1^H–^15^N HSQCs were recorded for samples selectively labelled with Leu, Phe and Ala/Lys, in the free and complex form. ^1^H–^15^N HSQCs were recorded for samples selectively unlabelled with Trp, Asn/Arg, Gln/Ile, Phe/Val and Lys/Leu. All data were processed using NMRPipe and NMRDraw (Delaglio et al. 1995) and analysed with CCPNMR Analysis (Vranken et al. 2005).

Titration experiments with ^2^H–^15^N labelled FGFR3 were carried out under the same conditions. Averaged chemical shift perturbations (CSPs) were calculated from the changes observed in chemical shifts between the apo FGFR3 spectrum and the FGFR3:PD 2:1 spectrum using the formula (Schumann et al. 2007):

$$\Delta {\updelta _{{\text{AV}}}}={\left[ {0.5 \times \left( {\Delta \updelta { ^1}{\text{H}}+0.2 \times \Delta \updelta { ^{15}}{\text{N}}} \right)} \right]^{1/2}}$$

### Assignment and data deposition

**Fig. 1:**
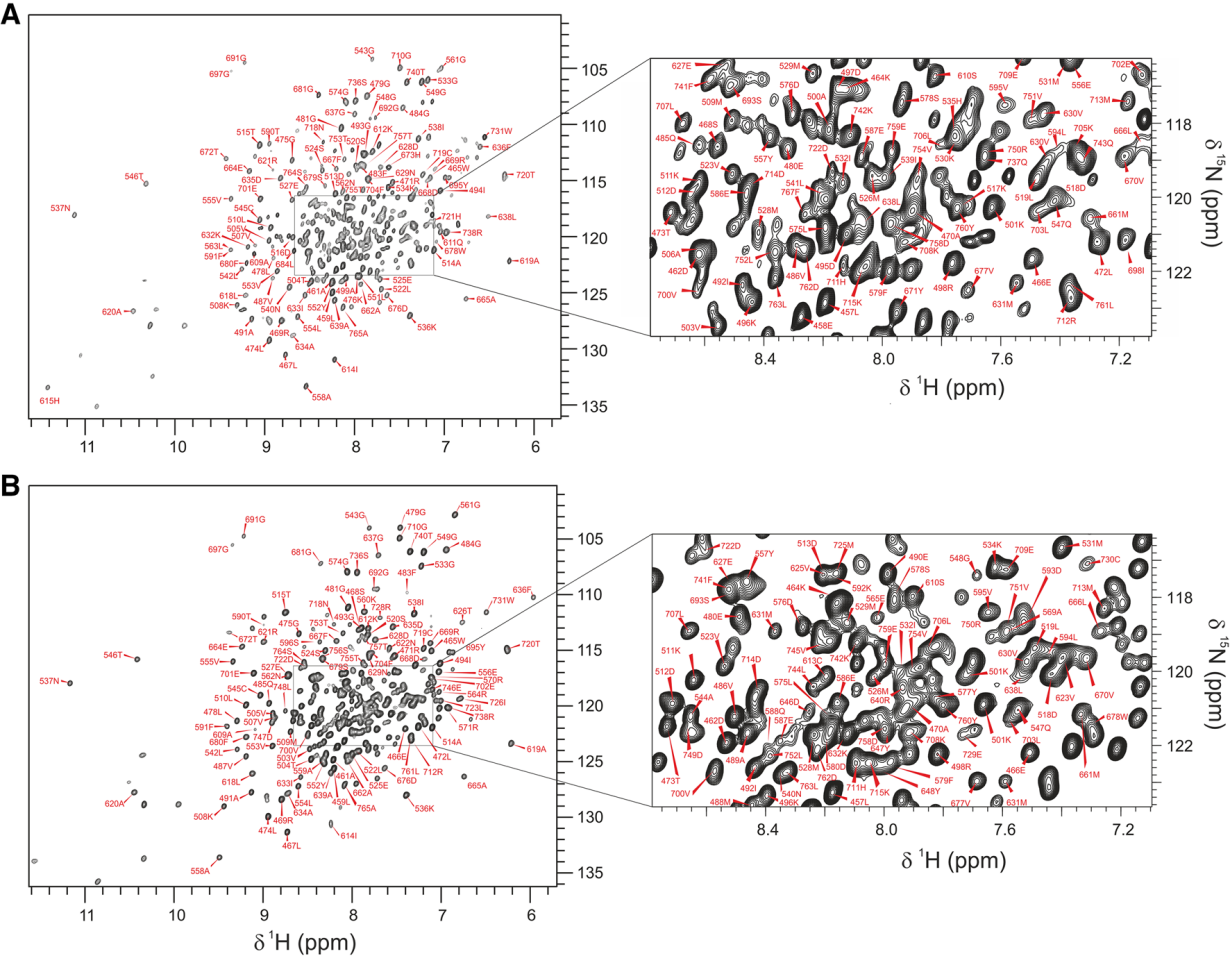
^1^H–^15^N TROSY–HSQC spectra of FGFR3. **a** Inhibitor-free protein in 50 mM PIPES-NaOH, 50 mM NaCl, 5 mM TCEP and 1 mM EDTA (pH 7.0) at 298 K; **b** PD-bound protein in the same buffer at 303 K. Resonances are labelled with the corresponding amino acid. On the right, magnified, central regions with crowded NMR resonances

**Fig. 2:**
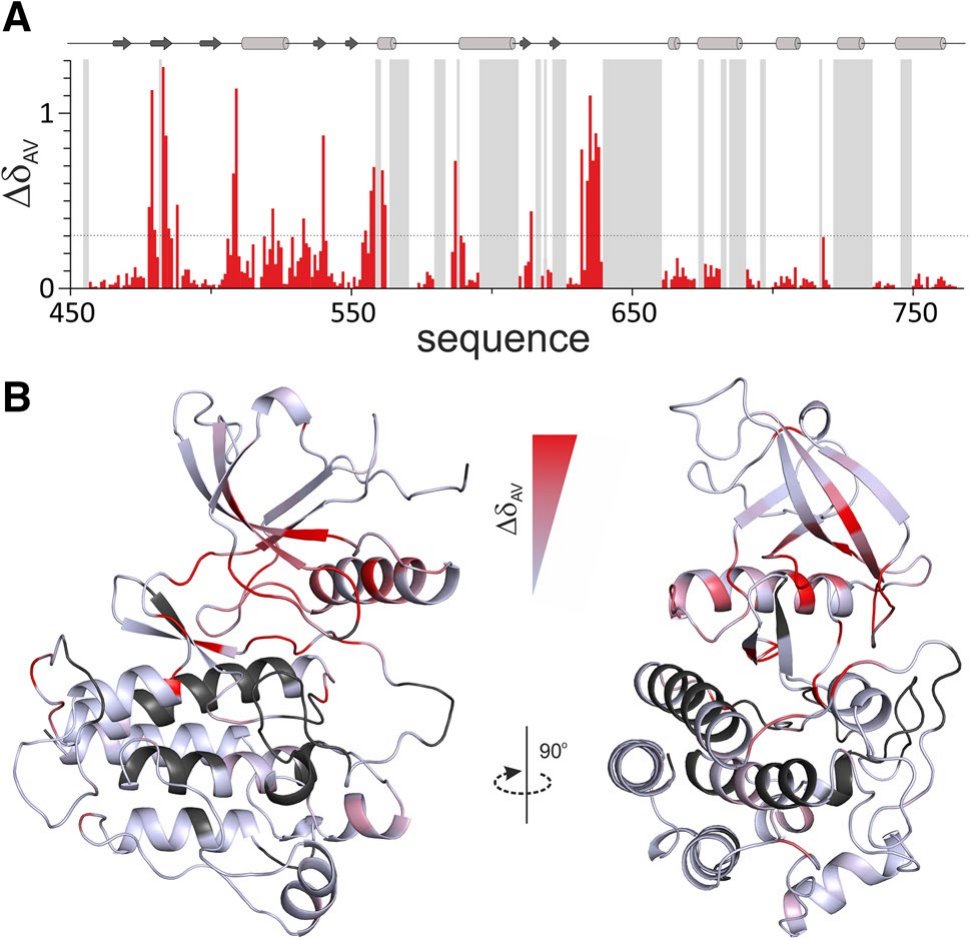
Chemical shift perturbations of FGFR3 upon PD173074 inhibitor interaction. **a** The weighted average of ^15^N and ^1^H_N_ chemical shift variation Δδ_AV_ = [0.5(Δδ H^2^ + 0.2 Δδ N^2^)]^1/2^ is reported as a function of the protein sequence. **b** Cartoon representation of FGFR3 structure [model of FGFR3 inactive/apo kinase generated with Modeller (Eswar et al. 2006) based on FGFR1 kinase-domain PDB ID: 4UWY], labelled by residue-specific amide chemical shift perturbation upon PD173074 binding on a red scale, (unassigned residues have a dark gray color): strong CSPs are observed in the N-terminal region (particularly the P-loop and hinge), and in the “DFG latch” clustered around the DFG Phe

The FGFR3 kinase domain is very challenging for NMR due to the low solubility, marginal stability and intrinsically dynamic nature of the protein, combined with its relatively large size (~ 35.4 kDa). The ^1^H–^15^N TROSY–HSQC spectra of free and PD-bound FGFR3 are shown in Fig. 1. Preliminary data indicated that FGFR3 in complex with PD was more stable than the inhibitor-free form; consequently, assignment of the complex was tackled first. 78% assignment of the backbone resonances (78% of HN and CB, 74% of N, CA and CO), of the PD-bound FGFR3 was achieved using a complete set of TROSY-based triple-resonance experiments (HNCA, HNCOCA, HNCACB, CBCACONH, HNCO, HNCACO) (Salzmann et al. 1998). Assignment of inhibitor-free FGFR3 backbone amide and carbon resonances was then accomplished by the use of a limited set of TROSY triple-resonance experiments (HNCA, HNCACB and HNCO) and by comparison to backbone assignments of free FGFR1 (Vajpai et al. 2014) and FGFR2 kinases (D. Cowburn, personal communication): both isoforms share more than 83% sequence identity with FGFR3 [calculated using Clustal Omega (Sievers et al. 2011)]. In addition, samples of free FGFR3 selectively labelled with Phe and Leu (U-^15^N-Phe, U-^15^N,^13^C-Leu) were used to solve ambiguities together with the assignment of PD-bound FGFR3. Ultimately, assignment of 75% of the backbone in the inhibitor-free form was achieved (comprising 75% HN and CA, 70% N, 69% CB and 68% CO); the kinase N-lobe is completely assigned apart from Ala 482, and the C-lobe partly assigned. Although extremely dynamic regions, such as the activation loop, have not been fully assigned (free FGFR3 residues 640–659 are completely missing, presumably due to conformational exchange broadening, while for the PD complex assignments for residues 645–648 are available), important residues such as the “DFG latch” [a hydrophobic cluster centred on the Phe of the DFG motif (Chen et al. 2017)] are assigned and present a large perturbation upon inhibitor binding (see Fig. 2). In general, numerous chemical shift changes were observed upon complex formation, most of which are located in the N-terminal region and at the interface between the two lobes. In particular, the P-loop and hinge region experience large CSPs, reflecting the direct contacts involving these parts of the kinase and the bound inhibitor. Although PD173074 itself is no longer in clinical development as an FGFR kinase inhibitor, it is representative of other so-called type I FGFR inhibitors that bind to the kinase in the ‘DFG-in’ conformational state of the activation loop, some of which are in late-stage clinical trials. Knowledge of the residues involved both in direct recognition of the inhibitor, as well as those within the allosteric network that experience perturbations on inhibitor binding, is paramount for future efforts to develop new-generation tyrosine kinase inhibitors that can exploit different conformational states of the enzyme. Backbone assignments have been deposited in the BioMagResBank database, with accession numbers 27082 for the inhibitor-free form and 27083 for the PD complex.
